# Health-related quality of life effects of enzalutamide in patients with metastatic castration-resistant prostate cancer: an in-depth post hoc analysis of EQ-5D data from the PREVAIL trial

**DOI:** 10.1186/s12955-017-0704-y

**Published:** 2017-06-23

**Authors:** Nancy Devlin, Michael Herdman, Marco Pavesi, De Phung, Shevani Naidoo, Tomasz M. Beer, Bertrand Tombal, Yohann Loriot, Cristina Ivanescu, Teresa Parli, Mark Balk, Stefan Holmstrom

**Affiliations:** 1 Office of Health Economics, Southside, 7th Floor, 105 Victoria Street, London, SW1E 6QT UK; 2 European Association for the Study of the Liver–Chronic Liver Failure (EASL-CLIF) Consortium, C/Mallorca 183, 08036 Barcelona, Spain; 3 Astellas Pharma Global Development, J.H. Oortweg 62, 2333 BE Leiden, The Netherlands; 4 Astellas Medical Affairs, Global Health Economic Outcomes Research (HEOR), 2000 Hillswood Dr, Chertsey, Surrey, KT16 0PS UK; 5 OHSU Knight Cancer Institute, Oregon Health & Science University, 3303 SW Bond Ave., CH14R, Portland, OR 97239 USA; 6 Institut de Recherche Clinique (IREC), Cliniques Universitaires Saint Luc, Av Hippocrate, 10 - 1200 Bruxelles – Belgique, Brussels, Belgium; 7 Institut Gustave Roussy, University of Paris Sud, 114, rue Edouard-Vaillant, 94805 Villejuif Cedex, France; 8 Quintiles, Siriusdreef 10 Beukenhorst Zuid 2132, WT Hoofddorp, The Netherlands; 9 Medivation, Inc., 525 Market St, Fl 36, San Francisco, CA 94105 USA; 10 Astellas Medical Affairs, Global HEOR, J.H. Oortweg 62, 2333 BE Leiden, The Netherlands

**Keywords:** Metastatic castration-resistant prostate cancer, Enzalutamide, Quality of life, Eq-5D

## Abstract

**Background:**

The effect of enzalutamide on health-related quality of life (HRQoL) in the PREVAIL trial in chemotherapy-naïve men with metastatic castration-resistant prostate cancer was analyzed using the generic EQ-5D instrument.

**Methods:**

Patients received oral enzalutamide 160 mg/day (*n* = 872) or placebo (*n* = 845). EQ-5D index and EQ-5D visual analogue scale (EQ-5D VAS) scores were evaluated at baseline, week 13, and every 12 weeks until week 61 due to sample size reduction thereafter. Changes on individual dimensions were assessed, and Paretian Classification of Health Change (PCHC) and time-to-event analyses were conducted.

**Results:**

With enzalutamide, EQ-5D index and EQ-5D VAS scores declined more slowly versus placebo and time to diverge from full health was prolonged. Average decline in EQ-5D index (−0.042 vs. –0.070; *P* < .0001) and EQ-5D VAS (−1.3 vs. –4.4; *P* < .0001) was significantly smaller with enzalutamide. There were significant (*P* < .05) between-group differences favoring enzalutamide in Pain/Discomfort to week 37, Anxiety/Depression at week 13, and Usual Activities at week 25, but no significant differences for Mobility and Self-care. The PCHC analysis showed more enzalutamide patients reporting improvement than placebo patients at weeks 13, 25, and 49 (all *P* < .05) and week 37 (*P* = .0512). Enzalutamide was superior (*P* ≤ .0003) to placebo for time to diverge from full health and time to first deterioration on Pain/Discomfort and Anxiety/Depression dimensions.

**Conclusions:**

This in-depth post hoc analysis showed that enzalutamide delayed HRQoL deterioration and had beneficial effects on several HRQoL domains, including Pain/Discomfort and the proportion of patients in full health, compared with placebo, and may help to support future analyses of this type.

**Trial registration:**

NCT01212991

## Background

Castration-resistant prostate cancer (CRPC) is associated with impaired health-related quality of life (HRQoL). This reflects, in part, the disease process itself [1–3], with HRQoL continuing to deteriorate as the disease progresses [2, 4]. In addition, treatment-related side effects can have a negative impact on HRQoL [2, 3]. While metastatic CRPC (mCRPC) had previously been associated with rapid disease progression and relatively short median survival (approximately 18 months) [2, 4], in recent years, several new treatments have shown a survival benefit [5]. Accordingly, as more patients receive treatment over longer periods, the importance of measuring HRQoL is increasingly recognized. For example, the Prostate Cancer Clinical Trials Working Group 2 (PCWG2) recommends serial assessment of HRQoL in clinical trials of treatments for progressive CRPC [6]. A number of tools are available to assess HRQoL in CRPC, including disease-specific questionnaires such as Functional Assessment of Cancer Therapy–Prostate (FACT-P) [7], cancer-specific questionnaires such as the European Organization for Research and Treatment of Cancer Quality of Life Questionnaire [8], and generic (i.e. non–disease-specific) questionnaires, such as the EQ-5D [9, 10].

Development of CRPC is due, in part, to sustained androgen receptor (AR) signaling despite castrate levels of testosterone [11, 12]. Thus, the AR is a key target for developing novel treatments for CRPC. Enzalutamide is a potent AR signaling inhibitor that impairs three stages of the AR signaling pathway: nuclear translocation of the receptor, DNA binding to AR response elements, and recruitment of co-activators [11]. It has a higher affinity for the AR than the first-generation agent, bicalutamide, and lacks partial agonist effects with higher AR expression [11]. Enzalutamide has been evaluated in both chemotherapy-treated (AFFIRM) and chemotherapy-naïve (PREVAIL) men with mCRPC. In both settings, it was associated with significant improvements in overall and radiographic progression-free survival compared with placebo [13, 14].

Patient HRQoL was assessed in the AFFIRM trial using FACT-P and the Brief Pain Inventory Short Form (BPI-SF) and in the PREVAIL trial using FACT-P and EQ-5D [13–16]. In AFFIRM, overall improvement in HRQoL (i.e. FACT-P total score) was reported by a greater proportion of patients receiving enzalutamide than those receiving placebo (42% vs. 15%; *P* < .0001) [16]. PREVAIL (NCT01212991) notably recruited patients with a relatively low symptom burden, and in addition to assessing FACT-P, it was the first randomized controlled trial to report EQ-5D responses in an exclusively chemotherapy-naïve mCRPC population [15].

EQ-5D outcomes from PREVAIL, such as change in EQ-5D index and visual analogue scale (VAS), were summarized by Loriot et al. [15]; however, only limited data were provided in that earlier paper as it reported on all HRQoL instruments included in PREVAIL (FACT-P, BPI-SF, and EQ-5D). A more in-depth exploration of EQ-5D dimensions can provide greater insight into drivers of change in the instrument’s two summary measures, particularly the EQ-5D index, and help reveal any patterns in those changes. Likewise, alternative methods to summarize change in the EQ-5D descriptive system, such as the Paretian Classification of Health Change (PCHC) [17], can help to identify further differences between treatments not explored in the earlier paper. Additionally, aspects such as the number of patients describing themselves in full health on the EQ-5D system can give a fuller picture of the nature of the study population and the effect of treatment over time. Such information can also be useful for planning future studies using EQ-5D in this population.

This paper, therefore, reports the results of a secondary analysis of data from the PREVAIL trial to provide greater insight into patterns of change on EQ-5D dimensions in PREVAIL and how they relate to changes on the EQ-5D index and VAS. This additional analysis should enhance understanding of the patient experience of treatment and provide useful information for those working with the EQ-5D in this field.

## Methods

### Study design and patients

Full details on the study design, patient eligibility criteria, and conduct of the study have been reported elsewhere [14]. Briefly, PREVAIL was a multinational, phase 3, randomized, double-blind, placebo-controlled trial. Patients aged ≥18 years were included if they had confirmed mCRPC, despite androgen-deprivation therapy, and were chemotherapy naïve. Additionally, patients had to be asymptomatic or mildly symptomatic (i.e. score of 0–3 on the Brief Pain Inventory Short Form questionnaire) with a good performance status (i.e. Eastern Cooperative Oncology Group [ECOG] Performance Status of 0 or 1). Treatment with oral enzalutamide (160 mg once daily) or placebo continued until the occurrence of unacceptable adverse events, confirmed radiographic progression, or a skeletal-related event warranting initiation of chemotherapy.

### EQ-5D questionnaire and outcomes

Patient HRQoL was assessed using the 3L version of EQ-5D, an international, standardized questionnaire for evaluating HRQoL [9, 10, 18]. It consists of five dimensions (Mobility, Self-care, Usual Activities, Pain/Discomfort, and Anxiety/Depression), each of which have three levels of problems (1, no problems; 2, some problems; and 3, extreme problems). It also includes the EQ-5D VAS to assess patients’ current health status (scale 0–100, where 0 = worse imaginable health state and 100 = best imaginable heath state). Results for the dimensions are combined to give a unique EQ-5D health state (or profile) for each patient, consisting of a five-digit code; for example, state 11111 indicates no problems in any dimension [18]. By applying weights derived from the general population [18, 19], health states can then be converted to a preference-weighted summary score, or EQ-5D index on which a score of 1 corresponds to full health, while 0 represents a state so bad it is considered equivalent to death. Thus, for both measures, higher scores indicate better HRQoL.

In the current study, EQ-5D was assessed at baseline and week 13, then every 12 weeks thereafter until drug discontinuation. EQ-5D index scores were derived by applying weights from the UK general population [20].

### Statistical analyses

EQ-5D questionnaire completion rates were calculated at each assessment time point by dividing the number of patients who answered all of the EQ-5D items by the total number of patients available (i.e. those who were still on study drug).

Analyses were performed on the intention-to-treat population, which included all patients who were randomized into the study, using data from all EQ-5D respondents. Results are presented up to week 61 only in view of the reduction in the effective sample size during follow-up (see Results).

Least squares mean (LSM) values (with 95% confidence intervals [CIs]) and changes from baseline in EQ-5D index and EQ-5D VAS scores (based on data from all time points) were estimated after adjusting by baseline EQ-5D index/EQ-5D VAS, age, fatigue score, pain score, and country in a mixed model for repeated measurements.

Average changes from baseline were estimated by dividing the cumulated change from baseline by the number of weeks of follow-up. For this analysis, LSM (standard error) and 95% CI of the difference between study arms in changes from baseline were estimated after adjusting by baseline EQ-5D index/EQ-5D VAS, age, fatigue score, pain score, and country in an analysis of covariance model.

For each EQ-5D dimension, the proportion of patients reporting no, some, or extreme problems was summarized at each time point. Additionally, the proportion of patients with an EQ-5D index score of 1 (full health) was summarized. EQ-5D data were also evaluated using the PCHC [17] whereby patients’ health was classified as: improved (improvement on at least one EQ-5D dimension and no worsening on any other dimension), worsened (deterioration on at least one EQ-5D dimension and no improvement on any other dimension), mixed (improved on at least one dimension and worsened on at least one other dimension), or no change. Finally, time-to-event analyses were used to estimate the benefit of enzalutamide compared to placebo in delaying or preventing deterioration in patients’ health. Inverted hazard ratios (1/HR) and 95% CIs were derived for the following endpoints using a Cox proportional hazards model: divergence from baseline “full health” (EQ-5D health state of 11111; this variable was applicable only to those patients in full health at baseline), first decrease on EQ-5D VAS, first worsening on PCHC, and first deterioration on selected dimensions (with Pain/Discomfort being of particular interest). The model was adjusted for the following baseline factors: age, fatigue score, pain score, geographic region, and baseline EQ-5D index or corresponding dimension value. The results can be interpreted as the reduction in each event rate for patients taking enzalutamide versus those receiving placebo.

An additional analysis explored the possibility of an association between changes on the Pain/Discomfort dimension of EQ-5D and changes on the Anxiety/Depression dimension. Odds ratios and 95% CIs were derived up to week 61. Data from patients in both study arms were aggregated for this analysis. A C statistic was used to summarize the strength of the relationship between Anxiety/Depression and Pain/Discomfort dimensions at each time point. For this statistic, a value close to 1 indicates a perfect association (i.e. in all cases, if Pain/Discomfort improves or worsens then so does Anxiety/Depression).

In all statistical analyses, significance is set at *P* < .05. No correction was made for multiple testing, since EQ-5D variables were considered as secondary, exploratory endpoints in the PREVAIL trial. This was a post hoc analysis and subsequent conclusions should take this into consideration.

## Results

In total, 1717 patients were randomized to receive enzalutamide (*n* = 872) or placebo (*n* = 845). Baseline demographic and disease characteristics, which have been described previously, were well balanced between treatment arms [14]. At baseline, mean (standard deviation) EQ-5D index scores (enzalutamide, 0.85 [0.15]; placebo, 0.84 [0.17]) and EQ-5D VAS scores (enzalutamide, 77.2 [16.7]; placebo, 75.9 [17.5]) were similar between groups. A similar proportion of patients in each group were in full health (index value = 1) at baseline (enzalutamide, 42.5%; placebo, 42.3%).

EQ-5D questionnaire completion rates exceeded 90.0% in both groups at all time points up to week 61. The number of patients available for EQ-5D assessment declined over time due to attrition of patients on study; study drug discontinuation was primarily due to disease progression. As expected, the attrition rate varied between treatment arms. Thus, by week 37 the number of patients with available EQ-5D dimension data had decreased from 857 at baseline to 671 in the enzalutamide group versus a reduction from 826 to 267 in the placebo group; by week 61 these numbers had decreased to 523 and 117 patients, respectively. Data on HRQoL was not collected after study drug discontinuation.

Analysis of individual EQ-5D dimensions (Table 1) showed that the effect of enzalutamide on HRQoL was primarily in the Pain/Discomfort dimension, with significant between-group differences (*P* < .05) to week 37. Data from individual EQ-5D dimensions also favored enzalutamide in Anxiety/Depression at week 13 (*P* = .006) and Usual Activities at week 25 (*P* = .03). There were no significant between-group differences in the Mobility and Self-care dimensions.

**Table 1:** Outcomes on individual EQ-5D dimensions by treatment group

	Baseline			Week 13			Week 25			Week 37			Week 49			Week 61		
EQ-5D dimensions	ENZA (*n* = 857), %	PBO (*n* = 826), %	*P* value	ENZA (*n* = 809), %	PBO (*n* = 624), %	*P* value	ENZA (*n* = 746), %	PBO (*n* = 367), %	*P* value	ENZA (*n* = 671), %	PBO (*n* = 267), %	*P* value	ENZA (*n* = 610), %	PBO (*n* = 176), %	*P* value	ENZA (*n* = 523), %	PBO (*n* = 117), %	*P* value
Mobility																		
Level 1	74.9%	74.7%	0.5940	74.7%	71.3%	0.3197	73.7%	70.8%	0.5823	70.4%	70.3%	0.8183	66.7%	30.9%	0.6454	68.5%	71.8%	0.7640
Level 2 or 3	25.1%	25.3%		25.4%	28.8%		26.3%	29.1%		29.7%	29.7%		33.3%	69.1%		31.6%	28.3%	
Self-care																		
Level 1	94.9%	93.8%	0.1726	93.9%	92.0%	0.2253	94.0%	90.6%	0.1034	92.4%	91.7%	0.2849	92.3%	92.0%	0.9186	6.9%	10.2%	0.0619
Level 2 or 3	5.1%	6.2%		6.1%	8.0%		6.0%	9.3%		7.7%	8.3%		7.7%	8.0%		93.1%	89.7%	
Usual Activities																		
Level 1	80.1%	80.5%	0.9685	74.9%	69.3%	0.0675	77.9%	71.8%	0.0262	76.2%	76.7%	0.3589	70.8%	71.8%	0.9266	72.7%	69.8%	0.6346
Level 2 or 3	20.0%	19.5%		25.1%	30.6%		22.1%	28.2%		23.9%	23.4%		29.3%	28.2%		27.31%	30.2%	
Pain/Discomfort																		
Level 1	55.8%	58.5%	0.4623	56.0%	47.8%	0.0051	59.8%	50.7%	0.0163	56.4%	47.6%	0.0475	52.6%	52.8%	0.6281	52.4%	53.9%	0.4483
Level 2 or 3	44.3%	41.5%		44.1%	52.2%		40.3%	49.4%		43.5%	52.5%		47.5%	47.1%		47.6%	46.2%	
Anxiety/Depression																		
Level 1	75.0%	72.9%	0.0690	74.4%	67.6%	0.0060	76.3%	71.0%	0.1610	75.9%	72.9%	0.5023	74.0%	68.8%	0.1762	70.8%	71.8%	0.7396
Level 2 or 3	25.0%	27.2%		25.6%	32.4%		23.8%	29.1%		24.1%	27.0%		26.0%	31.3%		29.2%	28.2%	

*ENZA* enzalutamide, *PBO* placebo

Differences refer to the comparison between groups in the percentage of patients with (2 or 3) or without (1) problems in each dimension. They do not take into account whether patients are in level 2 or 3. There were very few patients at level 3 in any dimension, even at 61 weeks. *P* values refer only to the between-group differences

**Fig. 1:**
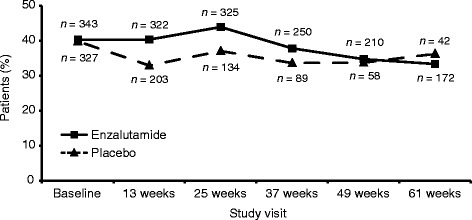
Number and proportion of patients in full health (EQ-5D state 11111) at each study visit, by study arm

Figure 1 shows the number and proportion of patients with an EQ-5D index value of 1 (full health) at different time points up to week 61. Although there was a tendency for the enzalutamide group to show a greater proportion of patients in health state 11111 up to week 37, the between-group difference was only statistically significant at week 13 (*P* < .05).

The PCHC analysis showed a greater proportion of enzalutamide patients reporting improvement than those receiving placebo (Table 2); between group differences were statistically significant (*P* < .05) at weeks 13, 25, and 49 and approached significance at week 37. A significantly (*P* < .05) greater proportion of placebo patients reported worsening up to week 25.

**Table 2:** Pareto classification of health change classification of changes from baseline in EQ-5D dimensions

	Enzalutamide (*n* = 872), n (%)	Placebo (*n* = 845), n (%)	*P* value
Week 13	*n* = 783 (89.8%)	*n* = 605 (71.6%)	
Worsening	208 (26.6%)	230 (38.0%)	<0.0001
No change	337 (43.0%)	242 (40.0%)	
Improvement	190 (24.3%)	94 (15.5%)	<0.0001
Mixed change	48 (6.1%)	39 (6.5%)	
Week 25	*n* = 726 (83.3%)	*n* = 356 (42.1%)	
Worsening	194 (26.7%)	123 (34.6%)	0.0078
No change	288 (39.7%)	151 (42.4%)	
Improvement	193 (26.6%)	64 (18.0%)	0.0018
Mixed change	51 (7.0%)	18 (5.1%)	
Week 37	*n* = 649 (74.4%)	*n* = 258 (30.5%)	
Worsening	198 (30.5%)	95 (36.8%)	0.0666
No change	252 (38.8%)	100 (38.8%)	
Improvement	146 (22.5%)	43 (16.7%)	0.0512
Mixed change	53 (8.2%)	20 (7.8%)	
Week 49	*n* = 594 (68.1%)	*n* = 168 (19.9%)	
Worsening	210 (35.4%)	61 (36.3%)	0.8192
No change	214 (36.0%)	74 (44.1%)	
Improvement	132 (22.2%)	22 (13.1%)	0.0093
Mixed change	38 (6.4%)	11 (6.6%)	
Week 61	*n* = 507 (58.1%)	*n* = 113 (13.4%)	
Worsening	193 (38.1%)	51 (45.1%)	0.1644
No change	180 (35.5%)	41 (36.3%)	
Improvement	101 (19.9%)	18 (15.9%)	0.3299
Mixed change	33 (6.5%)	3 (2.7%)	

Enzalutamide was statistically superior to placebo in the majority of the time-to-event analyses performed, including time to diverge from full health (*P* < .0001), time to first decrease in the EQ-5D Index or EQ-5D VAS (both *P* < .0001), time to first worsening on PCHC (*P* = .0003), and time to first deterioration on the Self-care (*P* = .0019), Pain/Discomfort (*P* < .0001), and Anxiety/Depression (*P* = .0003) dimensions (Table 3). Adjusted HRs for the analyses with statistically significant results ranged from 0.52 for any worsening from full health at baseline to 0.76 for worsening on the PCHC.

**Table 3:** Adjusted estimates of risk reduction of the onset of each deterioration assessment for patients taking enzalutamide vs. placebo (inverted hazard ratios [HR] and 95% confidence intervals [CI])

Deterioration events	Adjusted 1/HR	95% CI	*P* value
Any worsening from baseline full health (EQ index = 1)	0.52	0.42–0.65	<0.0001
Any decrease in EQ-5D index	0.53	0.48–0.61	<0.0001
Any decrease in EQ-5D VAS	0.62	0.55–0.70	<0.0001
“Worsening” on PCHC	0.76	0.65–0.88	0.0003
Worsening in EQ-5D dimensions			
Mobility	0.87	0.68–1.10	0.2357
Self-care	0.60	0.43–0.83	0.0019
Usual Activities	0.85	0.70–1.03	0.1066
Pain/Discomfort	0.57	0.47–0.68	<0.0001
Anxiety/Depression	0.66	0.53–0.83	0.0003

*PCHC* Paretian Classification of Health Change, *VAS* visual analogue scale

Relative risk reduction and 95% CIs from Cox proportional hazards models adjusted by the following baseline factors: age, fatigue score, pain score, geographic region, and baseline EQ-5D index or corresponding dimension value

Finally, results suggest that the risk of worsening in the Anxiety/Depression dimension increased substantially if there was worsening in the Pain/Discomfort dimension (Table 4). Conversely, the chance of improvement in the Anxiety/Depression dimension increased significantly if there was improvement in the Pain/Discomfort dimension. A C statistic analysis indicated a moderate association between improvement (C = 0.54–0.58) or worsening (C = 0.53–0.62) in Pain/Discomfort and improvement or worsening in Anxiety/Depression, respectively, during the study.

**Table 4:** Odds ratios for risk of worsening or improvement in EQ-5D anxiety/depression dimension when pain/discomfort dimension worsens or improves, respectively (not by treatment)

	Odds ratio (95% CI)				
	Week 13 (*n* = 1403)	Week 25 (*n* = 1087)	Week 37 (*n* = 914)	Week 49 (*n* = 770)	Week 61 (*n* = 625)
Worsening of Anxiety/Depression with worsening Pain/Discomfort	2.495 (1.749–3.559)	3.623 (2.426–5.410)	2.259 (1.431–3.564)	2.696 (1.690–4.303)	1.457 (0.832–2.554)
Improvement in Anxiety/Depression with improvement in Pain/Discomfort	2.514 (1.591–3.972)	1.890 (1.159–3.083)	2.937 (1.738–4.964)	2.916 (1.643–5.177)	2.789 (1.380–5.638)

*CI* confidence interval

## Discussion

The importance of assessing HRQoL in patients with advanced prostate cancer is increasingly recognized, and the PCWG2 recommends the use of validated questionnaires in clinical trials to characterize symptomatic outcome measures [6]. Patient HRQoL was thus included as a prespecified endpoint in the PREVAIL study [14]; these analyses provide important evidence on patients’ own views of their health, complementing objective measures such as overall survival and radiographic progression. Indeed, improved HRQoL has been linked to better clinical outcomes in mCRPC [21].

While many studies in mCRPC collect HRQoL data, to date this has been done using only disease-specific instruments. For example, it was previously shown that enzalutamide has HRQoL benefits relative to placebo in the post-chemotherapy setting using the FACT-P instrument [22]. However, few trials have examined HRQoL outcomes in chemotherapy-naïve patients with mCRPC [15, 23]. Moreover, PREVAIL was the first trial to use EQ-5D to assess HRQoL in chemotherapy-naïve mCRPC [15]. Although Loriot et al. [15] showed that, compared with placebo, enzalutamide significantly prolonged the time to deterioration in EQ-5D index and VAS scores, the earlier paper did not present results for EQ-5D dimensions, and so was unable to ascertain what was driving the changes in summary scores. To expand on Loriot et al. [15], we analyzed results primarily at the dimension and health-profile level and used different analytical approaches such as the PCHC, time-to-event analysis, and the number of patients reporting full health over time.

One advantage of this type of in-depth analysis is that the effects of treatment become clearer. For example, at first glance, Table 1 might be taken to indicate that patients remain relatively stable over the study period; e.g. no pain or discomfort was reported by 55.8% and 52.4% of patients in the enzalutamide group at baseline and at week 61, respectively. However, using the Paretian Classification of Change approach, when taking all dimensions together, there is considerable movement between levels of perceived problems, with outcomes favoring enzalutamide in terms of the proportion of patients improving and/or worsening, at all visits up to week 49. Simply presenting results as in Table 1, while useful in providing an overall picture of change at the dimension level, fails to capture all of the effects on EQ-5D. Likewise, Table 3 shows that time to deterioration was consistently worse in the placebo group. This type of information can help provide a more complete picture of patient-reported outcome results. Indeed, time-to-event analyses indicate the protective effect of enzalutamide; for instance, the adjusted HR for "moving away from baseline full health" of 0.52 means that patients taking enzalutamide have about half the probability of worsening from baseline full health compared to those receiving placebo over the study period. In other words, for this outcome, the deterioration event rate in the enzalutamide arm is approximately half that of the placebo group.

The secondary analysis of data from PREVAIL reported here shows that, compared with placebo, enzalutamide was associated with significant HRQoL benefits in some but not all EQ-5D dimensions and that the delayed deterioration in overall HRQoL, as measured by the EQ-5D index and EQ-5D VAS, was largely driven by changes in the dimensions of Pain/Discomfort and Anxiety/Depression. There was no significant difference between enzalutamide and placebo groups in the Mobility and Self-care dimensions. Furthermore, PCHC analysis showed that the proportion of patients with improvements in HRQoL was significantly greater with enzalutamide than with placebo at the majority of time points.

Pain is one of the most prominent and debilitating symptoms for patients with mCRPC and skeletal metastases [24]. In large clinical trials of patients with progressive CRPC, approximately 35% of patients had substantive pain [6]. Pain is associated with a significant reduction in HRQoL [1] and is a significant predictor of survival in mCRPC [25, 26]. However, pain is often under-treated in patients with cancer [27, 28], and in clinical trials in progressive prostate cancer early changes in pain are often not acted upon without other evidence of disease progression [6]. The current analysis indicates that, based on data from the EQ-5D Pain/Discomfort domain, enzalutamide exhibited beneficial effects on pain and discomfort relative to placebo. Thus, the proportion of patients with no problems in this dimension was greater with enzalutamide than placebo from week 13 through to week 37, with a significant difference between groups during these time points. Additionally, time to first deterioration in the Pain/Discomfort dimension was significantly longer for enzalutamide. These results are consistent with the recommendation from the PCWG2 that effective treatments should delay and/or prevent HRQoL deterioration associated with disease progression, as well as delay the onset of significant pain in men with progressive CRPC [6]. They also support previous analyses from the PREVAIL study showing that enzalutamide had beneficial effects on pain, as measured using the Brief Pain Inventory Short Form [15].

We have also seen evidence of a beneficial effect of enzalutamide relative to placebo on anxiety and depression, with data from the EQ-5D Anxiety/Depression dimension favoring enzalutamide at week 13 and a significant delay in the time to first deterioration in Anxiety/Depression with enzalutamide. Depression and anxiety are two of the most common psychological symptoms in patients with cancer and are associated with poorer treatment outcomes, increased hospitalization, and higher mortality rates [29]. In a recent meta-analysis of data from 27 articles in 4494 men with prostate cancer, the prevalence of clinical anxiety and depression was approximately 15% during treatment [29]. However, evidence regarding psychological distress and effective psychological interventions for men with mCRPC is sparse; for example, the anxiety experienced varies greatly depending on individual circumstances [3]. Also, a favorable prostate-specific antigen response can alleviate anxiety in men with prostate cancer.

Interestingly, the PREVAIL results suggest a moderate association between Pain/Discomfort and Anxiety/Depression, with the observation that a worsening or improvement in the Pain/Discomfort dimension was associated with a corresponding increase in the risk of worsening or improvement, respectively, in Anxiety/Depression in these patients with chemotherapy-naïve mCRPC. This is not unexpected as depressive and anxiety disorders have been shown to be associated with worse pain severity over time [30], and pain has been shown to significantly correlate with depression in patients with advanced cancer [31]. We would hypothesize that at least some of the Anxiety/Depression benefits observed are linked to effective control of disease.

In the large global patient population included in the current study, baseline EQ-5D index scores were slightly higher (indicating better health) than in other studies in patients with mCRPC [2, 32–34], which might reflect the treatment (chemotherapy-naïve) and symptom (asymptomatic or mildly symptomatic) status of patients enrolled in PREVAIL. In fact, an interesting finding of this additional analysis is the high proportion of patients who report being in full health (i.e. EQ-5D health state 11111) at baseline. A recent analysis of EQ-5D-3L data from the 2012 Health Survey for England showed that, of the total sample of 7294 respondents from the general population, 56% reported full health [35], compared with approximately 42% at baseline in the current study. However, in the present study, most patients (79%) were ≥65 years of age; for the male population in the 2012 Heath Survey for England, only 19% were aged >65 years. Also of note was the high number of respondents in the enzalutamide group reporting full health at the final visit (*n* = 172/523 [33%] enzalutamide patients remaining in the study at week 61). Only 42 patients in the placebo group reported being in full health at week 61, although that represented 40.4% of the remaining patients (*n* = 117) receiving placebo, a number attributable to much higher rates of attrition.

The main limitation of our study was the high attrition rate, particularly in the placebo group, as a result of disease progression. This was expected, but it did limit the analyses due to the reduced sample size at later time points and the imbalance between groups in the numbers of patients. However, for time-to-event analysis, the attrition had less impact if the events happened, whereas the impact was actually greater on the time point analysis. It should also be noted that since attrition is nonrandom, after the majority of patients are off study drug, those left may comprise a subgroup of patients whose disease progresses slowly, even with placebo, or responds particularly well to the active treatment, thereby potentially biasing the results of analysis in favor of the placebo arm. Thus, analysis of those patients remaining on study suggested little or no difference between groups on any EQ-5D dimension toward the end of the study (week 61).

Another limitation of our analysis is the lack of HRQoL data after treatment discontinuation. However, patients are likely to be treated with other agents post-discontinuation, which could confound interpretation of the results. Finally, the nature of the patient population (high level of functioning; ECOG Performance Status of 0 or 1; asymptomatic/mildly symptomatic) may not reflect the real-world population of men with mCRPC, which limits the generalizability of the results. Further epidemiological research could show the extent to which the PREVAIL study population is representative of the mCRPC population seen in clinical practice.

## Conclusions

In conclusion, in this population of patients with chemotherapy-naïve mCRPC from the PREVAIL trial, this post hoc analysis of EQ-5D data at the dimension level showed that enzalutamide was associated with significant benefits in terms of Pain/Discomfort and Anxiety/Depression compared with placebo and may help to support future analyses of this type. There was some evidence that benefits in the Anxiety/Depression dimension were associated with patient evolution in the Pain/Discomfort dimension, but further investigation is required. Also of note was the relatively high proportion of patients reporting full health on EQ-5D both at baseline and end of study, with proportions that are likely similar to those reporting full health in general population samples; this suggests that enzalutamide can help substantial numbers of patients with mCRPC to maintain a quality of life approaching that of similarly aged samples of the general population.

## Abbreviations

AR: Androgen receptor
CI: Confidence interval
CRPC: Castration-resistent prostate cancer
ECOG: Eastern Cooperative Oncology Group
EQ-5D VAS: EQ-5D visual analogue scale
FACT-P: Functional assessment of cancer therapy–prostate
HR: Hazard ratio
HRQoL: Health-related quality of life
LSM: Least squares mean
mCRPC: Metastatic castration-resistent prostate cancer
PCHC: Paretian classification of health change
PCWG2: Prostate Cancer Clinical Trials Working Group 2.

## References

[1] Sandblom G, Carlsson P, Sennfält K, Varenhorst E (2004). A population-based study of pain and quality of life during the year before death in men with prostate cancer. Br J Cancer.

[2] Sullivan PW, Mulani PM, Fishman M, Sleep D (2007). Quality of life findings from a multicenter, multinational, observational study of patients with metastatic hormone-refractory prostate cancer. Qual Life Res.

[3] Payne H, Pearcy R (2012). Symptoms and health-related quality of life in castration-resistant prostate cancer: the patient's perspective. J Mens Health.

[4] Merseburger AS, Bellmunt J, Jenkins C (2013). Perspectives on treatment of metastatic castration-resistant prostate cancer. Oncologist.

[5] Suzman DL, Antonarakis ES (2014). Castration-resistant prostate cancer: latest evidence and therapeutic implications. Ther Adv Med Oncol.

[6] Scher HI, Halabi S, Tannick I, Morris M, Sternberg CN, Carducci MA (2008). Design and end points of clinical trials for patients with progressive prostate cancer and castrate levels of testosterone: recommendations of the prostate cancer clinical trials working group. J Clin Oncol.

[7] Esper P, Mo F, Chodak G, Sinner M, Cella D, Pienta KJ (1997). Measuring quality of life in men with prostate cancer using the Functional assessment of cancer therapy-prostate instrument. Urology.

[8] Aaronson NK, Ahmedzai S, Bergman B, Bullinger M, Cull A, Duez NJ (1993). The European Organization for Research and Treatment of cancer QLQ-C30: a quality-of-life instrument for use in international clinical trials in oncology. J Natl Cancer Inst.

[9] EuroQol Group (1990). EuroQol--a new facility for the measurement of health-related quality of life. Health Policy.

[10] Rabin R, de Charro F (2001). EQ-5D: a measure of health status from the EuroQol group. Ann Med.

[11] Tran C, Ouk S, Clegg NJ, Chen Y, Watson PA, Arora V (2009). Development of a second-generation antiandrogen for treatment of advanced prostate cancer. Science.

[12] Hu R, Denmeade SR, Luo J (2010). Molecular processes leading to aberrant androgen receptor signaling and castration resistance in prostate cancer. Expert Rev Endocrinol Metab.

[13] Scher HI, Fizazi K, Saad F, Taplin ME, Sternberg CN, Miller K (2012). Increased survival with enzalutamide in prostate cancer after chemotherapy. N Engl J Med.

[14] Beer TM, Armstrong AJ, Rathkopf DE, Loriot Y, Sternberg CN, Higano CS (2014). Enzalutamide in metastatic prostate cancer before chemotherapy. N Engl J Med.

[15] Loriot Y, Miller K, Sternberg CN, Fizazi K, De Bono JS, Chowdhury S (2015). Effect of enzalutamide on health-related quality of life, pain, and skeletal-related events in asymptomatic and minimally symptomatic, chemotherapy-naive patients with metastatic castration-resistant prostate cancer (PREVAIL): results from a randomised, phase 3 trial. Lancet Oncol.

[16] Fizazi K, Scher HI, Miller K, Basch E, Sternberg CN, Cella D (2014). Effect of enzalutamide on time to first skeletal-related event, pain, and quality of life in men with castration-resistant prostate cancer: results from the randomised, phase 3 AFFIRM trial. Lancet Oncol.

[17] Devlin NJ, Parkin D, Browne J (2010). Patient-reported outcome measures in the NHS: new methods for analysing and reporting EQ-5D data. Health Econ.

[18] EuroQol 2015. EQ-5D-3L User Guide. https://euroqol.org/wp-content/uploads/2016/09/EQ-5D-3L_UserGuide_2015.pdf. Accessed 13 Jan 2016.

[19] Pickard AS, Neary MP, Cella D (2007). Estimation of minimally important differences in EQ-5D utility and VAS scores in cancer. Health Qual Life Outcomes.

[20] Dolan P (1997). Modeling valuations for EuroQol health states. Med Care.

[21] Sullivan PW, Nelson JB, Mulani PM, Sleep D (2006). Quality of life as a potential predictor for morbidity and mortality in patients with metastatic hormone-refractory prostate cancer. Qual Life Res.

[22] Cella D, Ivanescu C, Holmstrom S, Bui CN, Spalding J, Fizazi K (2015). Impact of enzalutamide on quality of life in men with metastatic castration-resistant prostate cancer after chemotherapy: additional analyses from the AFFIRM randomized clinical trial. Ann Oncol.

[23] Basch E, Autio K, Ryan CJ, Mulders P, Shore N, Kheoh T (2013). Abiraterone acetate plus prednisone versus prednisone alone in chemotherapy-naive men with metastatic castration-resistant prostate cancer: patient-reported outcome results of a randomised phase 3 trial. Lancet Oncol.

[24] Gater A, Abetz-Webb L, Battersby C, Parasuraman B, McIntosh S, Nathan F (2011). Pain in castration-resistant prostate cancer with bone metastases: a qualitative study. Health Qual Life Outcomes.

[25] Armstrong AJ, Garrett-Mayer E, Yang Y-CO, Carducci MA, Tannock I, de Wit R (2007). Prostate-specific antigen and pain surrogacy analysis in metastatic hormone-refractory prostate cancer. J Clin Oncol.

[26] Halabi S, Vogelzang NJ, Kornblith AB, Ou SS, Kantoff PW, Dawson NA (2008). Pain predicts overall survival in men with metastatic castration-refractory prostate cancer. J Clin Oncol.

[27] Deandrea S, Montanari M, Moja M, Apolone G (2008). Prevalence of undertreatment in cancer pain. A review of published literature. Ann Oncol.

[28] Apolone G, Corli O, Caraceni A, Negri E, Deandrea S, Montanari M (2009). Pattern and quality of care of cancer pain management. Results from the cancer pain outcome research study group. Br J Cancer.

[29] Watts S, Leydon G, Birch B, Prescott P, Lai L, Eardley S (2014). Depression and anxiety in prostate cancer: a systematic review and meta-analysis of prevalence rates. BMJ Open.

[30] Gerrits MM, van Marwijk HW, van Oppen P, van der Horst H, Penninx BW (2015). Longitudinal association between pain, and depression and anxiety over four years. J Psychosom Res.

[31] Ko HJ, Seo SJ, Youn CH, Kim HM, Chung SE (2013). The association between pain and depression, anxiety, and cognitive function among advanced cancer patients in the hospice ward. Korean J Fam Med.

[32] Skaltsa K, Longworth L, Ivanescu C, Phung D, Holmstrom S (2014). Mapping the FACT-P to the preference-based EQ-5D questionnaire in metastatic castration-resistant prostate cancer. Value Health.

[33] Wolff JM, Donatz V, Klier J, Erhardt W, Dass RN, Geiges G (2012). Quality of life among German patients with metastatic castration-resistant prostate cancer [abstract]. Value Health.

[34] Wu EQ, Mulani P, Farrell MH, Sleep D (2007). Mapping FACT-P and EORTC QLQ-C30 to patient health status measured by EQ-5D in metastatic hormone-refractory prostate cancer patients. Value Health.

[35] Feng Y, Devlin N, Herdman M (2015). Assessing the health of the general population in England: how do the three- and five-level versions of EQ-5D compare?. Health Qual Life Outcomes.

